# Role of Actin Dynamics and *GhACTIN1* Gene in Cotton Fiber Development: A Prototypical Cell for Study

**DOI:** 10.3390/genes14081642

**Published:** 2023-08-18

**Authors:** Adnan Iqbal, Sibgha Aslam, Mukhtar Ahmed, Fahad Khan, Qurban Ali, Shiming Han

**Affiliations:** 1 School of Biological Sciences and Technology, Liupanshui Normal University, Liupanshui 553004, China; aadiiq@yahoo.com; 2 Plant Breeding and Acclimatization Institute—National Research Institute, Radzikow, 05-870 Blonie, Poland; 3 Government Boys College Sokasan, Higher Education Department, Azad Jammu and Kashmir, Bhimber 10040, Pakistan; 4 Department of Plant Protection, Faculty of Agricultural Sciences, Ghazi University, Dera Ghazi Khan 33001, Pakistan; 5 Department of Plant Breeding and Genetics, Faculty of Agricultural Sciences, University of the Punjab, Lahore 54590, Pakistan

**Keywords:** cotton fiber, actin dynamics, *GhACTIN1*, plant actin, gene pyramiding

## Abstract

Cotton crop is considered valuable for its fiber and seed oil. Cotton fiber is a single-celled outgrowth from the ovule epidermis, and it is a very dynamic cell for study. It has four distinct but overlapping developmental stages: initiation, elongation, secondary cell wall synthesis, and maturation. Among the various qualitative characteristics of cotton fiber, the important ones are the cotton fiber staple length, tensile strength, micronaire values, and fiber maturity. Actin dynamics are known to play an important role in fiber elongation and maturation. The current review gives an insight into the cotton fiber developmental stages, the qualitative traits associated with cotton fiber, and the set of genes involved in regulating these developmental stages and fiber traits. This review also highlights some prospects for how biotechnological approaches can improve cotton fiber quality.

## 1. Introduction

Cotton is grown worldwide owing to its high economic value. More than 90 percent of natural fiber is produced from cotton, and it is cultivated in almost 80 countries worldwide. Cotton is considered a cash crop that feeds 250 million people around the globe according to the US National Council of Textile Organizations (NCTO). In 2017, the US earned USD 77.9 billion by producing and processing cotton fiber, textile, and clothes shipments, and these industries employed 550,500 workers (http://www.ncto.org/2018-state-of-the-u-s-textile-industry-address/, accessed on 22 March 2018). In Pakistan, cotton is thought to be the lifeline of the economy on account of its 0.8% share in the GDP and its 4.5% value added to agriculture. During the fiscal year 2018–2019, Pakistan produced 9.86 million bales (Economic Survey of Pakistan 2018–2019).

## 2. Qualitative Traits of Cotton Fiber

Cotton fiber is generally called an epidermal outgrowth of seed originating from an ovule [1]. Cotton is one of the most significant natural sources of textile-grade fiber, which, in its mature form, is made up of >90% cellulose [2,3]. The value of a cotton crop increases if the cotton fiber has better qualitative traits, such as whiteness, staple length, micronaire value, strength, and uniformity index [4].

### 2.1. Fiber Length

The fiber length can be determined by measuring the fiber while still attached to the seeds [5]. Recently, with the advent of technology, fiber length has been measured using the photoelectric method. This technology uses ginned fiber fixed in combs and passed through photoelectric scanners [6]. Based on its length, upland cotton is divided into four categories: fiber having a length less than 21 mm is defined as short, 22 to 25 mm is medium, 26 to 28 mm is medium to long, and 29 to 34 mm is considered long [7].

### 2.2. Fiber Strength

Fiber strength is a vital textile trait that is significant in fabrics. Modern technologies like open-end rotor spinning operating systems require higher fiber strength [8]. Two methods have been reported for measuring fiber strength: the Pressley apparatus and the Stelometer [9]. Both of these methods use a bundle of a certain weight of a fiber fixed into clamp jaws followed by force applied until the breakage of the fiber bundle. The breakage force represents the fiber strength [10]. Generally, the fiber strength is denoted as g/tex, which is the force in grams required to break apart one tex unit (1000 m of fiber) of fiber bundle.

### 2.3. Micronaire Value

The micronaire value is considered a commercially important trait. It is regarded as an indirect measure of fiber maturity and fineness. Usually, low micronaire values are considered better. The micronaire value (mic) is measured by air permissibility through a fiber sample enclosed in a fixed-dimension container [11,12]. Upland cotton ranges from 3.5 to 4.9 mic, while a 3.7 to 4.2 mic value is regarded as a premium range [13]. The mic value is important for the better spinning of yarn, while maturity enhances the dyeing quality of the fiber [14].

### 2.4. Fiber Maturity

**Figure 1:**
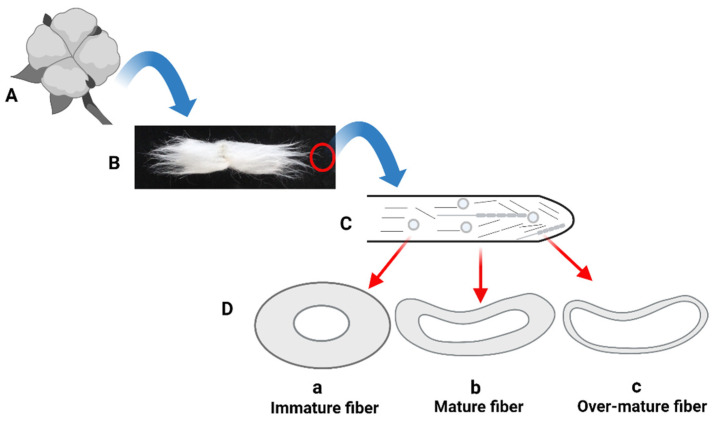
Schematic diagram of cotton fiber characteristics: (**A**) A mature cotton boll containing seeds and attached fiber; (**B**) A single seed attached with lint fiber; (**C**) An illustration of a developing single fiber cell showing different proteins and actin filaments; (**D**) Attributes of different levels of fiber maturation; (**a**) Immature fiber produces low yield, weak fiber and more of neps; (**b**) Mature fiber produces higher yield, strong fiber, and fewer neps; (**c**) Over-mature fiber produces higher yield and strong fiber but produces weak yarn (image drawn by the authors).

Fiber maturity, a commercial term, cannot be confused with fiber maturation, usually defined as the chronological series of time taken from anthesis (day of flowering) to the harvesting of mature fiber. Fiber maturity is defined as the ratio of the cell wall thickness to the diameter of cell wall thickness compared to the size of the lumen [11,12], and based on the fiber’s diameter, different attributes, such as immature, mature, and over-mature fiber, are given (Figure 1). In terms of diameter, upland cotton fibers range from 21 to 29 µm; however, finer fibers have a range of diameter from 17 to 20 µm [12]. Fiber maturity is another important trait when dealing with cotton fibers in the textile industry. Normally, a 0.7 to 0.9 maturity value is regarded as a moderate range for the smooth processing of cotton fiber. The immature cotton fiber, having a value of less than 0.7, does not make good yarn, produces neps in ginning, and is prone to breakage during spinning, while over-mature fiber, having a value of more than 0.9, produces rough yarn, which is undesirable for users [15].

### 2.5. Fiber Fineness

Fiber fineness constitutes the fiber perimeter, diameter, linear density, cross-section area, and uniformed surface. Fiber fineness is mostly associated with linear density. Fine fiber makes the yarn stronger than yarn made up of short and rough fiber [16]. Fiber fineness can be defined as the measure of the unit mass in micrograms (μg) per unit length (inches) of fiber to evaluate the linear density [17].

## 3. Cotton Fiber Development Stages

Cotton fiber is an elongated single-celled assembly that originates from a seed coat. The fiber develops in four separate yet over-lapping stages known as: (1) Initiation; (2) Elongation; (3) Secondary cell wall synthesis; and (4) Maturation [18]. A fully mature fiber following elongation and cell wall synthesis may reach 6 cm long [19].

### 3.1. The Initiation Stage of Cotton Fiber

Fiber initiation is the most important stage in cotton yield. The number of fibers initiated at this stage will determine the total fiber per ovule leading to the final yield. The day of anthesis is considered 0 DPA, and fiber initiation lasts from 0 DPA to almost 5 DPA [20,21]. Previous studies have revealed that a broad range of genes is involved in fiber initiation. Some of the important genes which regulate either fiber initiation or both initiation and elongation are listed in Table 1.

### 3.2. Role of Phytohormones in Fiber Initiation

Phytohormones also regulate fiber development. Acid indol-3-acetic (IAA) is a naturally existing auxin and has been reported to have a vital role in fiber development [30,31]. Previous reports have revealed that either the exogenous application of auxins or over-expression of auxin gene promotes fiber initiation and increases cotton yield and fiber quality [31,32]. A recent study by Zhao et al. [21] reported that the exogenous application of IAA to the cotton plants showed a higher number and greater fiber size at 0 DPA in experimental plants compared to non-treated (control) plants. Consequently, experimental plants showed a higher yield of cotton fiber and quality of fiber uniformity, strength, staple length, and micronaire value.

### 3.3. Cotton Fiber Elongation Stage

Elongation is the second stage of fiber development which starts after initiation. During elongation, fiber cells elongate because of higher intracellular turgor pressure and cell wall relaxation [33,34]. The advances in molecular studies of cotton fiber have reported many genes involved in fiber elongation and cell wall synthesis [35]. Plant hormones also have an effective role in controlling fiber elongation; ethylene production is involved in controlling fiber elongation by regulating sucrose-synthase, expansins, and tubulin-related genes. It has been reported that abscisic acid also inhibits fibre growth in cotton ovules [36]. A few of the important genes involved in regulating fiber elongation are listed in Table 2.

### 3.4. Secondary Cell Wall Synthesis

The elongation phase in developing fiber is followed by secondary cell wall synthesis. Many genes have been reported to be involved in the synthesis of secondary walls. Although work on identifying and isolating such genes started very late, much progress has been made until now. The secondary wall synthesis phase usually lasts from 25 to 40 DPA but remains until cotton bolls dehiscence, i.e., 50–60 DPA [46]. Very few genes that are strictly involved in secondary wall synthesis have been identified. Most of these genes are reported to have a combined role in fiber elongation and wall synthesis [47]. Phytosterols also regulate the fiber development process at elongation and secondary wall synthesis. A recent study by Niu et al. [48] showed that the over-expression of the *GhSMT2–1* gene changes the phytosterol level. Compared to control plants, the increased level of sitosterol and reduced level of campesterol in GhSMT2–1 over-expressed transgenic cotton plants resulted in shorter but thicker fiber. The results implied that a higher level of sitosterol or a lower level of campesterol inhibits fiber elongation but promotes secondary wall thickening. The important genes involved in regulating fiber elongation and secondary wall synthesis are listed in Table 3.

### 3.5. Cotton Fiber Maturation Stage

**Figure 2:**
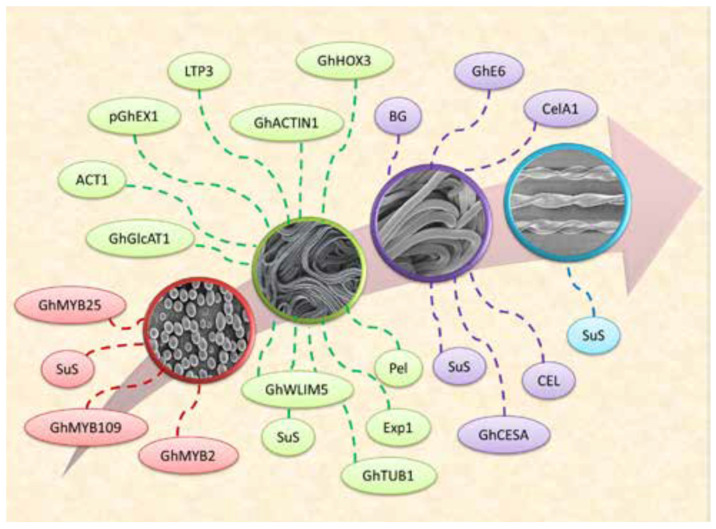
Genes involved in development of cotton fiber [56].

Fiber maturation is the final phase of its developmental stages. Fiber maturation can be directly equated to fiber quality. Immature fiber is weak, has a low yield, high neps, and shows uneven dyeing with poor spinning quality, while mature fiber is strong and has good cotton fiber yield, lower neps, and good dyeing and spinning properties. Overly mature fiber also has strong fiber with high yield, weaker yarn, good dyeing, and poor spinning qualities [2]. Cotton varieties can be categorized into early, middle, and late maturity cultivars based on the required fiber maturation time. Upland cotton showing early maturity is associated with the plant’s early boll opening feature and short architecture [54]. Because of the small plant architecture and high-density planting early maturity varieties are gaining importance in China. However, early maturity cotton plants have lower cotton fiber yields than the middle and late-maturity varieties [55]. Latif et al. [56] explained the cotton fiber development and some important genes related to each developmental stage, as shown in Figure 2.

## 4. Role of *ACTIN* Genes in Cotton Fiber Development

Cotton fiber development largely depends on cell wall biosynthesis and cytoskeleton arrangement. Cytoskeleton dynamics control many cellular processes, such as the movement of organelles, cell wall formation, and cell division. Microfilaments (actin-filament), microtubules, and intermediate filaments are the main constituents of the cytoskeleton [57]. In most cells, actin filaments are involved in secretory vesicle transportation to the cell membrane and cell wall, enhancing cell expansion. The actin cytoskeleton also regulates tip growth and cell elongation. Dozens express actin proteins to hundreds of genes in the *ACTIN* family. *Arabidopsis* has 10 actin genes, of which 8 are functional, and 2 are categorized as pseudogenes, while cotton plants have been identified with 16 actin genes [45].

### 4.1. Actin Filament Development Pathway and Actin-Binding Proteins (ABPs)

Actin is expressed in monomeric form, which is known as G-actin. The G-actin polymerises form a filament known as F-actin [58]. The formation of actin filaments by monomeric actin includes nucleation, polymerization/capping, and F-actin bundling and cross-linking activities. Many Actin-Binding Proteins (ABPs) are divided according to their association among G-Actin-binding/G-actin capping proteins and F-Actin regulators (ABPs), which are involved in either polymerization or depolymerization, and proteins that serve to crosslink and/or bundle the actin microfilaments [59]. The detailed pathway and the ABPs are involved in every step of actin microfilament formation.

#### 4.1.1. Nucleation of Actin Filaments

Nucleation is the first step in the formation of a new filament. The nucleation of actin microfilaments is not a spontaneous process; rather, it depends on many nucleating proteins. Polymerization is energetically hostile unless actin monomers are nucleated. During polymerization/capping, actin filaments form two ends; a pointed end, which is slow growing, and a barbed end, which is fast growing. Arp2/3 complex is one of the first known nucleating molecules, which caps at a pointed end and makes the barbed end available for polymerization. However, WASP homology2 domains of WASP protein, SCAR-WAVE protein, and verprolin/WIP have also been very important for the Arp2/3 complex in actin nucleation [59,60]. Foramins have also been found in actin filament nucleation, apart from the Arp2/3 complex. The formin proteins and Spire also nucleate actin polymerization. However, studies indicated that the foramin homology2 (FH2) domain dimer stretches to accommodate the progressive addition of actin monomers to the barbed end of a filament [61].

#### 4.1.2. Polymerization and Capping of Microfilament

**Figure 3:**
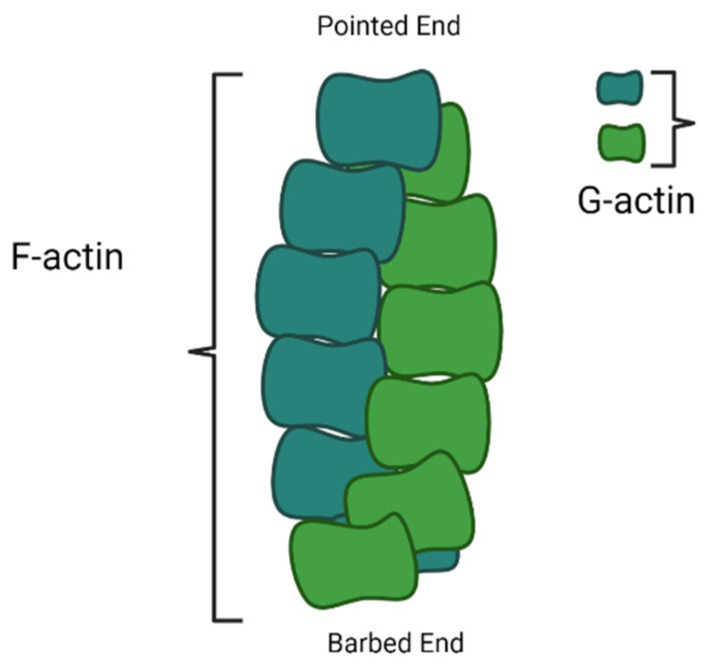
Diagram showing polymerization of F-actin filament on barded end (image drawn by the authors).

After nucleation, the microfilaments start growing by adding actin monomers at the barbed end, also known as the growing end; however, the pointed end is a non-growing end (Figure 3). The FH2 domain of formins directly nucleates actin monomers to form actin filaments by protecting growing ends from capping proteins while guiding the rapid insertion of new actin subunits. Residues Ile1431 in the knob and Lys1601 and Lys1359 in the post of the FH2 domain of Bni1p, a formin protein, manifest as actin-binding sites. The FH1 domain of formins not only recruits profilin–actin complexes but also accelerates filament elongation at least five times faster than the rate of diffusion-limited subunit addition at the free barbed ends of filaments [62]. The process of actin filament development happens at the non-growing pointed end.

Once the filaments grow enough, the length is controlled by many proteins, usually called cappers proteins, such as gelsolins and tensins, which inhibit the addition of further monomers. Adding cappers proteins at the pointed ends reduces the monomer loss and facilitates the extension of microfilament [63].

The polymerized filament (F-actin) can be depolymerized by Actin Depolymerized Factor (ADF)/Cofilin. The depolymerization activity of ADF/Cofilin complex is further enhanced by Actin Interaction Protein-1 (AIP-1) [59]. In contrast to the filament, the depolymerization protein, tropomyosin, and nubuline have been identified to stabilize the actin filament in muscle cells. Several other proteins have poly-proline-motifs filament stabilization by recruiting polymerization machinery [61].

#### 4.1.3. F-Actin Bundling and Cross Linking

After actin F-filament formation, the next step is actin bundling which is carried out by the alignment of the F-filament (F-actin) in a parallel or anti-parallel manner. Actin filament bundling is usually accomplished by proteins with two actin-binding domains [61]. The arrangement of bundled actin-filaments into orthogonal arrays is further mediated by proteins having multi-actin-binding domains. Actin-binding proteins, involved in cross-linking processes, have two or many domains, usually separated by a spacer. Filamin (dimeric) or spectrin (tetrameric) proteins cross-link. A monomeric protein called tansgelin has also been reported to be involved in cross-linking [64].

#### 4.1.4. Plant LIM, an Actin-Bundling Protein

**Figure 4:**
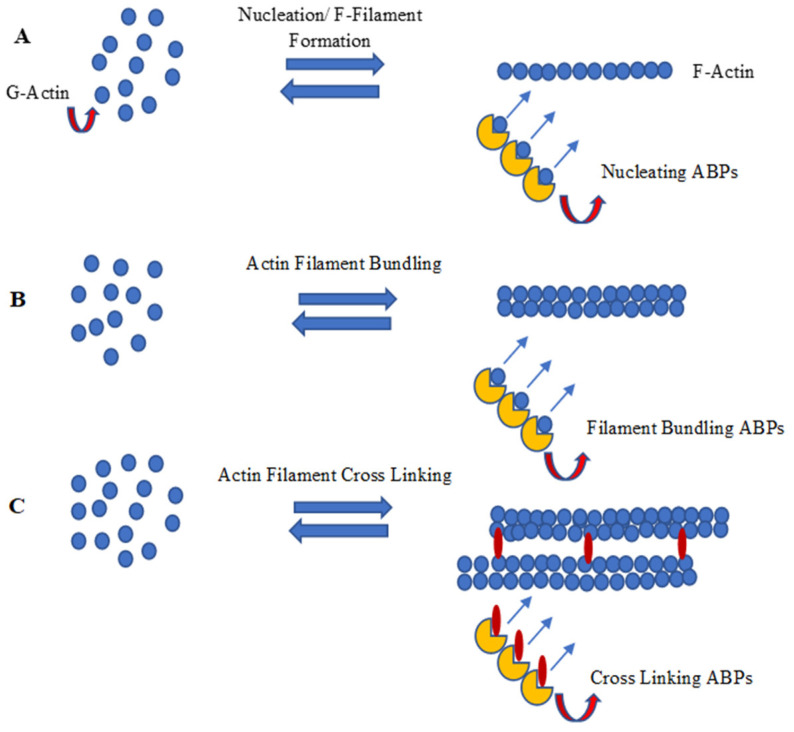
Schematic diagram of (**A**) nucleation, (**B**) polymerization/capping and (**C**) F-actin bundling and cross linking (image drawn by the authors).

Plant LIM proteins are another important class of ABPs [65], which are found to be dispersed in the cytosol and nucleoplasm. The LIM-domain-containing proteins in the nucleus are preferentially involved in tissue-specific gene regulation and determination of cell fate, whereas the cytoplasmic LIM-domain-containing proteins are involved mainly in cytoskeletal organization [66]. The term “LIM” originates from the initials LIN-11, ISL-1, and MEC-3, the first proteins observed to contain this particular homeodomain. Following this, all proteins containing LIM domains are called LIM proteins or LIM domain-containing proteins [67]. Most of the LIM proteins have two different LIM domains, each comprising 55 amino acids [68] and having the broad consensus sequence (CX2CX16-23HX2C)X2(CX2CX15-30CX2C/H/D) in which eight cys-his conserved residues form two zinc finger projections. The motifs in LIM domains are involved in protein–protein interactions and possess conserved scaffolds that recognize a diverse variety of target proteins. Each Zinc-finger motif within the LIM domains contains two Zinc coordinating cys-residues which assist in forming a β hairpin connection with the target protein. In LIM2, the single LIM domain consists of two Zinc fingers with a core of bulky hydrophobic residues [69]. Phylogenetic analyses of plant LIM proteins separate them into seven classes (XLIM1, WLIM1, WLIM2, βLIM1, PLIM1, PLIM2, and PLIM2-like) [70]; or into six categories, in which GhLIM1, GhWLIM2, and GhWLIM5 belong to the WLIM2 subgroup. Bioinformatics analysis shows that GhWLIM2 and GhWLIM5 have strong evolutionary relationships [41]. In cotton, there are many LIM-domain-containing proteins that modulate actin filament bundlings, such as GhPLIM1, which is predominantly involved in anther development [67], and WLIM1a, which is involved in fiber elongation along with secondary wall synthesis [49]. Cotton WLIM1a contains two domains: Domain 1 (D1) is involved in actin-bundling activity, whereas Domain 2 (D2) participates in DNA binding [65]. Figure 4 summarizes nucleation, polymerization/capping, and F-actin bundling and the cross-linking process of actin filament development through a schematic diagram.

## 5. Classification and Function of Plant Actin

The actin cytoskeleton in plants has a pivotal role in regulating cellular morphogenesis. In plants, the actin cytoskeleton controls many specialized cell functions such as root hairs, pollen tubes, trichomes, and stomatal guard cells [71,72]. The actin exists in two forms: un-polymerized molecules as G-Actin and polymerized filaments as F-Actin [58]. The genome of *Arabidopsis thaliana* contains a total of 10 actin genes, excluding 2 pseudogenes [45]; another 8 actin genes fall into 2 classes—encoding vegetative and reproductive protein isovariants [73]. The vegetative isovariants predominate in the stems, roots, leaves, and petals, whereas reproductive isovariants are found in pollen, ovules, and embryonic parts [74,75]. The *ACTIN-1* gene, from the reproductive class, is involved in fiber elongation [45], while other *ACTIN* genes from the reproductive part, such as *ACTIN4/12* class, are expressed in premature and mature pollen, vascular tissues, and tapetum, whereas *ACTIN12* expression was noted in the pericycle during lateral root initiation [76]. *ACTIN11*, one of the distinctive reproductive genes, is expressed in roots, and its misexpression leads to a change in the morphology of roots and trichomes [74]. A three-dimensional (3D) model of the ACTIN-1 protein of *A. thaliana* indicates that it possesses four subdomains, similar to the sub-domains observed in mammalian actin. However, in subdomain 2, the DNase-I loop presents most of the variable parts [73]. The monomeric actin (G-actin) molecule has four domains that bind ATP in their centers, thereby triggering polymerization. The hydrolysis of ATP leads to conformational changes in these domains, making this ADP-actin molecule susceptible to depolymerisation from the actin filament [61].

## 6. Role of *GhACTIN1* Gene in Cotton Fiber Development

Although Arabidopsis is the model plant in which actin genes have been well studied and characterized, the role of the actin gene in cotton still needs to be explored further for the identification and manipulation of the potential genes involved in fiber development. Plant actin is considered to be conserved at the gene level; however, divergence occurred on a protein–structural level during evolution. Li et al. [45] reported that 16 actin genes deduced from cotton have grouped/diverged into 9 sub-groups compared to 6 groups of Arabidopsis, and the variation in *GhACT* genes occurred more notably [75].

*GhACTIN1* gene was found to be expressed predominantly during fiber elongation. The cloning of the 0.8 kb promoter of *GhACTIN1*, taken from the 5′ upstream region, fused with the GUS marker transformed in cotton, showed GUS activity in fiber and validated the role of the *GhACTIN1* gene in fiber development regulation. No or very low GUS activity in the stem, root, leaf sepals, and petals reflects the least expression of the *GhACTIN1* gene in these tissues, which reflects its lack of role in these tissues [45]. The transcript level of *GhACTIN1* during fiber elongation (8–14 DPA) reaches its highest level of gene expression and is gradually reduced in the later fiber developmental stages [45]. Furthermore, actin turnover during fiber development is vital to keep the process uninterrupted. The RNAi inhibition of *GhACTIN1* in cotton fiber drastically reduced the F-actin filaments network; consequently, fiber length and strength were found to be reduced, which suggested that the *GhACTIN1* gene has a major role in fiber elongation, but the contribution of other genes, such as *GhACTIN2* and *GhACTIN5*, cannot be completely ruled out [45].

### Regulation of Fiber Elongation by Interaction between Cotton-Annexin (Gbanx6) and ACTIN1

Actin dynamics are regulated by many ABPs, such as ADF and profiling [77,78]. GhPFN-2 is a profilin and is expressed in developing cotton fiber during elongation. The over-expression of GhPFN-2 terminates the elongation phase prematurely and shows the early start of secondary wall synthesis; as a result, fiber length decreased significantly. Abundant F-actin filaments were also observed during the elongation phase [50,51]. Previous studies have validated that profiling, such as GhPFN-2, ADF, and related ABPs regulating actin dynamics by Ca^2+^ stimulation [79]. Annexins (a multigene family) are considered Ca^2+^-dependent or Ca^2+^-independent ABPs. These annexins are cytoskeleton and membrane-phospholipids binding proteins in many eukaryotic cells [80,81,82].

Plant cells have almost 0.1% annexin protein. These proteins are found in the membrane, cytoplasm, and cell wall [83,84]. Annexins are active in cell signaling and control material movement across the cell membrane as they can bind with Ca^2+^ and interact with membrane lipids [85,86]. Plant cell annexins accumulate at the tip of root hairs along with pollen tubes growing cells [87,88], and this localization of annexins facilitatess cell polar growth. Due to the binding capability of annexins to Ca^2+^ and lipid membranes, they were studied in cotton plants for their potential role in fiber elongation [89]. Elongating fibers have been reported to have 3–5 times higher fatty acid (mostly sphingolilids) content compared to ovules [47]. Huang et al. [76] revealed that cotton annexin anxGb6 interacts with fiber GbAct-1, a fiber specific actin, and plays an important role in fiber elongation.

## 7. Biotechnological Approach of Genetic Transformation to Improve Cotton Fiber

The success of the textile industry depends on the perpetual availability of fiber which could fulfill modern industry demands. There is no doubt that fiber obtained from local cotton is not of the necessary quality to meet the needs of the textile industry, especially for its fineness, staple length, strength, and maturity index. Synthetic fiber is the biggest contemporary challenge to natural fiber, as it can provide all the parameters in demand by the textile industry. Cotton crops are complex, sensitive, and susceptible to abiotic and biotic stresses like insect pests, CLCuV (cotton leaf curl virus), and weeds [90,91,92]. Lepidopterans insects alone, including pink bollworms, armyworm, and spotted bollworm, account for 30% of the total losses of cotton crop quality and significantly decrease its quantity [90]. Cotton Leaf Curl Virus is a devastating challenge to the cotton crop. The reduced yield consequently affects lint percentage (GOT %), fiber fineness and maturity, fiber length, strength, and maturity index [93,94]. Weeds, among all of these, are the single biggest threat, accounting for 47.5% of the total losses of the cotton crop and affecting the yield during the initial weeks of growth [92]. Cotton yield is highly influenced by abiotic stresses such as salt, drought, and temperature [95]. Although cotton crop is considered salt tolerant [96], morphological characters like plant height, number of stem nodes and internodes, the number of fruiting branches, and biomass notably affect salinity [97].

Similarly, drought and insufficient water availability cause a significant reduction in cotton yield [98,99]. High temperature is another important abiotic stress to the cotton crop and causes a decrease in fertilization efficiency [100], pollination [101,102], and boll size [103]. Fiber length is also reported to be reduced when the canopy temperature (*T*_C_) is raised above 31 °C [104].

It is important to address the challenges mentioned above, as any threat to cotton crops indirectly threatens the textile industry. Strategies have been devised to reduce these challenges’ losses and enhance the overall growth and fiber characteristics. Classical breeding has been used to improve the cotton yield and fiber with improved qualities by crossing a good cultivar with another suitable cultivar. This strategy comes with some limitations, such as the fact that only a limited gene pool can be among the suitable cultivars, and adding novel traits from other organisms becomes difficult through simple breeding [40,105].

Biotechnology is a way forward in modern times to achieve the required characteristics in an organism. Cotton fiber provides a good model for studying cell elongation and cell wall biosynthesis using biotechnological approaches [19]. Improved fiber yield and quality can be achieved through genetic modification. The idea of the over-expression of a certain gene to achieve the preferably required characteristic has become widespread, such as in fiber elongation, as reported by Zhang et al. [106], through the over-expression of *GhFIM-2.* FIM (Fimbrin) are the actin-bundling proteins vital in pollen-tube growth in lily and *Arabidopsis* [107,108]. The over-expression of *GhFIM-2* from FIM family enhances the actin filament bundling at the fiber elongation stage and helps propel the secondary wall biosynthesis. Thus, this indicates the role of GhFIM-2 in fiber development by actin dynamic re-arrangement [106].

The over-expression of GhPFN-2, a profilin, in cotton fibers results in secondary cell wall synthesis initiation by terminating the elongation phase before the time. This early termination of the elongation phase and early onset of secondary wall synthesis resulted in a short length of cotton fibers compared to the wild type. Thicker F-actin bundles at the elongation stage reorient the fiber bundles from the transverse to the oblique position. Before the microtubule’s re-orientation, F-actin abundance proved an essential trigger to switch from the elongation phase to secondary wall synthesis [51]. SPS (sucrose phosphate synthase) is important in the sucrose synthesis pathway. The catalyzation of fructose-6-phosphate into sucrose is carried out by SPS enzyme and has a role in fiber development [109]. Cotton fiber constitutes >90% of the cellulose. Cellulase synthase is also a significant enzyme that controls cellulose biosynthesis and plays an important role in determining fiber strength [110]. Biosynthesis and transport of VLCFA (Very Long Chain Fatty Acids) are reported to be very important in regulating fiber development. The over-expression of *AKR2A* (ankyrin repeat-containing protein 2A), an Arabidopsis gene, in cotton plants revealed that it promotes the elongation of cotton fiber by increasing the VLCFA contents in transgenic-lines compared to non-transgenic. The *AKR2A* gene also promotes fiber elongation by the signaling of hydrogen-peroxide. The results shows that *AKR2A* is a potential candidate gene for increasing cotton fiber yield as well as quality using a genetic engineering approach [38].

Improved cotton fiber yield and quality can be achieved using fiber-specific promoters that control the targeted gene expression in fibers. However, limited investigations have been carried out on fiber-specific promoters. To explore the molecular basis of cotton fiber development, Hou et al. [111] reported that *GhSCFP* (*Gossypium hirsutum* seed coat and fiber-specific protease) expression was higher during fiber initiation and elongation. The fiber specificity of the promoter was investigated in transgenic cotton and tobacco plants and confirmed by cloning the 5′ upstream region of GhSCFP, fused with the GUS and GFP markers.

Besides over-expression, gene knockdown approaches using CRISPR/Cas technology is another approach to improve cotton yield and fiber quality [112]. However, to meet the demands of the textile industry, instead of a single approach, scientists should use a combinational approach that combines all the possible technologies, such as gene over-expression, gene knockdown, and molecular breeding for crop improvement in a short time span [113].

## 8. Conclusions

Cotton fiber quality is a multigenic trait, which can be improved through in depth knowledge and targeted application in order to introduce many features by gene pyramiding. The current review gives an insight into how to improve cotton fiber quality using biotechnological approaches to meet the demands of the textile industry. Also, it explores the role of actin dynamics focusing on the *ACTIN1* gene of *G. hirsutum*.

## Figures and Tables

**Table 1:** List of genes involved in regulation of cotton fiber initiation and elongation.

Gene	Function	Reference
*GhPIN3a*	Fiber initiation	[22]
*GhMYB2*	Fiber initiation	[23]
*GaMYB2*	Fiber initiation	[24]
*GhMYB2*	Fiber initiation	[25]
*GhHD1*	Fiber initiation	[26]
*GhE6*	Fiber initiation	[27]
*GhSuS*	Fiber initiation and elongation	[28]
*GhPIN1a_Dt, GhPIN6_At* and *GhPIN8_At*	Fiber initiation and elongation	[29]

**Table 2:** List of genes involved in regulation of cotton fiber elongation.

Gene	Function	Reference
*SuS*	Fiber elongation	[37]
*AKR2A*	Fiber elongation	[38]
*GhCaM7*	Fiber elongation	[39]
*WLIM5*	Fiber elongation	[40,41]
*GhCFE1A*	Fiber elongation	[42]
*GhHOX3*	Fiber elongation	[43]
*GhPEPC1, GhPEPC2*	Fiber elongation	[44]
*GhACTIN1*	Fiber elongation	[40,45]
*GhEXP1*	Fiber elongation	[34]

**Table 3:** List of genes involved in regulation of cotton fiber elongation and secondary wall synthesis.

Gene	Function	Reference
*GhSMT2–1*	Secondary wall synthesis	[48]
*WLIM1a*	Fiber elongation and secondary wall synthesis	[49]
*GhADF1*	Fiber elongation and secondary wall synthesis	[50]
*GhPFN2*	Fiber elongation and secondary wall synthesis	[51]
*CelA1*	Fiber elongation and secondary wall synthesis	[52]
*GhEF1A*	Fiber elongation and secondary wall synthesis	[53]
